# Evaluation of the three-in-one team-based care model on hierarchical diagnosis and treatment patterns among patients with diabetes: a retrospective cohort study using Xiamen’s regional electronic health records

**DOI:** 10.1186/s12913-017-2705-2

**Published:** 2017-11-28

**Authors:** Xuejun Li, Zhibin Li, Changqin Liu, Junfeng Zhang, Zhonghai Sun, Yuji Feng, Jing Mei, Chengming Gu, Xiaoying Li, Shuyu Yang

**Affiliations:** 1 Department of Endocrinology and Diabetes, the First Affiliated Hospital, Xiamen University, Xiamen, China; 2 Xiamen Diabetes Institute, No.55 Zhenhai Road, Xaimen, 361003 China; 3 Epidemiology Research Unit, the First Affiliated Hospital, Xiamen University, Xiamen, China; 4 Lianqian Community Health Service Center, Xiamen, China; 5 Department of information, Xiamen Health and Family Planning Commission, Xiamen, China; 6 Pfizer Investment Co., Ltd, Beijing, China; 7 IBM Research China, Beijing, China; 8 Department of Endocrinology, Zhongshan Hospital, Fudan University, Shanghai, China

**Keywords:** Health policy reform, Chronic disease, Policy evaluation, Hierarchical health care

## Abstract

**Background:**

Xiamen is a pilot city in China for hierarchical diagnosis and treatment reform of non-communicable diseases, especially diabetes. Since 2012, Xiamen has implemented a program called the “three-in-one”, a team-based care model for the treatment of diabetes, which involves collaboration between diabetes specialists, general practitioners, and health managers. In addition, the program provides financial incentives to improve care, as greater accessibility to medications through community health care centers (CHCs). The aim of this study was to evaluate the effectiveness of these policies in shifting visits from general hospitals to CHCs for the treatment of type 2 diabetes mellitus (T2DM).

**Method and materials:**

A retrospective observational cohort study was conducted using Xiamen’s regional electronic health record (EHR) database, which included 90% of all patients registered since 2012. Logistic regression was used to derive the adjusted odds ratio (OR) for patients shifting from general hospitals to CHCs. Among patients treated at hospitals, Kaplan-Meier(KM) curves were constructed to evaluate the time from each policy introduction until the switch to CHCs. A k-means clustering analysis was conducted to identify patterns of patient care-seeking behavior.

**Results:**

In total, 89,558 patients and 2,373,524 visits were included. In contrast to increased outpatient visits to general hospitals in China overall, the percentage of visits to CHCs in Xiamen increased from 29.7% in 2012 to 66.5% in 2016. The most significant and rapid shift occurred in later periods after full policy implementation. Three clusters of patients were identified with different levels of complications and health care-seeking frequency. All had similar responses to the policies.

**Conclusions:**

The “three-in-one” team-based care model showed promising results for building a hierarchical health care system in China. These policy reforms effectively increased CHCs utilization among diabetic patients.

## Background

Rapid demographic and economic changes have made non-communicable diseases (NCD) the number one health problem in China, contributing to more than 80% of the country’s 10·3 million annual deaths and nearly 70% of its total disease burden [1, 2]. Diabetes is a major contributor to the NCD burden. A nationally representative sample survey of Chinese adults in 2010 found that the estimated prevalence of diabetes was 11.6% and the prevalence of prediabetes was 50.1% [3]. Geographic prevalence of diabetes and the medical resources to treat and prevent it are vary [3, 4].

In China, primary health care facilities, mainly community health centers (CHCs) and their outpost clinics, and tertiary care facilities, including general hospitals (GHs) and specialty hospitals, operate independently and compete for patients. Due to low trust in clinics and CHCs, patients often seek care at large hospitals even for simple health problems including routine chronic disease management of NCDs [5, 6]. Use of tertiary care facilities for routine chronic disease management can lead to a waste of medical resources and low overall quality of medical services patients received [6, 7]. It may also be ineffective for individual patients, who have reported higher satisfaction with the health care they received in CHCs [8].

In response to the challenges of NCDs, the Chinese government initiated comprehensive health care reforms nationwide in 2009. The five major goals of the health care reform included: 1. expanding insurance coverage to more than 90% of the population; 2. establishing a national essential drug list (EDL) to meet basic medication needs; 3. improving the primary health care delivery system to provide primary health care and to manage referrals to specialist care and hospitals; 4. making public health services available and equal for all; 5. piloting public hospital reforms [2, 5, 9, 10]. To improve the population’s health, and to effectively manage health care costs in the long-term, a key goal is to establish a hierarchical diagnosis and treatment system for the monitoring and prevention of chronic diseases and improvement of the community health service system [9].

Health policy research and evaluation are important components of the health care reform. Using health care networks and the database information created by the reforms, health policy evaluation is ongoing nationwide and in experimental sites throughout the country [11]. In 2014, the National Health and Family Planning Commission (NHFPC) selected the city of Xiamen as the pilot city for hierarchical diagnosis and treatment reform for diabetes and hypertension. As a municipality in the economically-developed southeast coastal area of China, Xiamen has a relatively high level of health care, and the average life expectancy is above 80 years. Chronic disease, however, is one of the main challenges to public health in Xiamen. The prevalence of hypertension and diabetes among residents in Xiamen is 16.67% and 4.61% respectively, and these two diseases were the first and second highest ranked causes of medical visits for the four consecutive years before 2014 [12]. During the full year of 2014 (January 1st–December 31st), more than 50% of outpatient visits in tertiary hospitals in Xiamen were due to chronic diseases, and more than 30% of these visits were for renewing an existing prescription.

Since 2012, Xiamen instituted a series of reform measures intended to encourage more appropriate health care-seeking behavior in patients with chronic diseases and more efficient use of health care resources. Reforms include upgrading infrastructure, information and communication technology in community health care facilities, establishing a clearly defined role for primary health care, improving incentives for health care providers for implementing chronic disease management in CHCs, creating an insurance payment plan, and expanding the essential drug list. Based on the experience with these reforms, in 2014 Xiamen introduced a “three-in-one” team-based care model for chronic disease management, experimented first among patients with hypertension and diabetes [7]. In this model, patients with diabetes or hypertension are assigned to a team of three healthcare workers that consisting of a specialist from a general hospital (GH), a general practitioner (GP) from a CHC, and a dedicated health manager. Together, the triad team is responsible for managing each patient’s health by means of health education and routine clinical follow-ups.

The aim of this study was to evaluate the effect of Xiamen health policy reform on health care utilization among diabetes patients. To determine the effectiveness of the policy reforms, this research evaluated the proportions of patient visits to general hospitals vs. CHCs, and the dynamic switch patterns from general hospitals to CHCs, for treatment of T2DM.

## Methods

### Data source

**Fig. 1:**
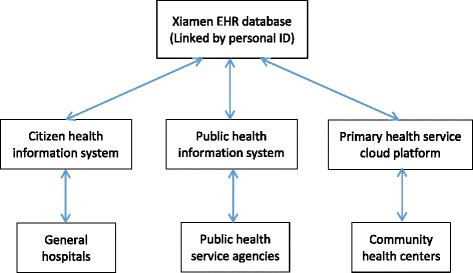
Schematic description of the Xiamen EHR database

The data source for this study was the retrospective regional electronic health record (EHR) observational database in Xiamen, which was established in 2006 and designed to cover all medical institutions in the city, including general hospitals, CHCs, and public health sectors. The database included 90% of the residents of Xiamen registered since 2012. The EHR were systematically collected from all city medical facilities and centralized by the Health and Family Planning Commission to form a regional health care database (Fig. 1). Patient visits to different facilities were under a unique patient ID, which made it possible to track individual medical records.

All health system reform policies related to the “three-in-one” team-based care model were summarized by interviewing the officials from Xiamen Health and Family Planning Commission (Table 1).

**Table 1:** Summary of policies for hierarchical medical system in Xiamen

	Time	Category			Policy change/Intervention
		A^*^	B^**^	C^***^	
P_0_	08/11	NA			Reference period
P_1_	08/12	x			Establishment of “Hospital-Community” integrated service model
	10/12			x	Increase of Essential Drug List (EDL) for hypertension and diabetes to 48 types
				x	Extension of the prescription interval at community health centers to 1 month
P_2_	01/14		x		Financial incentives to GPs for disease management at an average of ¥ 10,000/GP/year
		x			Initiation of “1 + 1 + X” model: Paired specialists from tertiary hospitals with GPs from community health centers
P_3_	10/14		x		Financial incentives for disease management increased to an average of ¥ 40,000/GP/year
		x			Initiation of “three-in-one” model: added health managers to specialist/GP pairs
		x			Establishment of diabetes patients network for enhanced disease management
				x	Increase of EDL for hypertension and diabetes to 84 types
P_4_	04/15	x			Establishment of the regional hierarchical medical system for chronic disease management, structured around an “overall health care network for diabetic and hypertensive patients”: • Full implementation of the “three-in-one” model • Enhanced information system for diabetic and hypertensive patients • Enhanced two-way patient referral system between GHs and CHCs
			x		Change in the financial incentive to 600¥/person/year for diabetes patients and 300¥/person/year for hypertension patients
		x			Pilot in closing out-patient service in tertiary hospitals
P_5_	07/15	x			Formal establishment of health manager position in CHCs
				x	Full implementation of the same EDL in community health centers as in general hospitals
				x	Extension of the prescription interval at community health centers to 2 months
			x		Change of periodical budget payment for CHCs to immediate payment settlement, and removal of the reimbursement limitations for CHCs
P_6_	01/16		x		Removal of upper limit on financial incentives to GPs for disease management

*A: Hospital-CHC integration; **B: Financial incentives to CHC/GPs; ***C: Drug availability in CHC

### Inclusion criteria

A retrospective cohort was constructed using de-identified patient records registered in the regional EHR database. All medical records of patients with diabetes treated in the index period between August 1st, 2012, and March 31st, 2016 were included. The index event was defined asT2DMrelated diagnosis, prescription for T2DM medication, or both occurring at any time on or after August 1st, 2012 until March 31st, 2016.

Patients were also included if they had made at least one medical visit within 360 days before the index event. Patients were excluded if they were diagnosed with gestational diabetes or type I diabetes, were under 18 years old, or if their gender was unknown in the pre-index period.

### Outcome measures

The main objective of this study was to evaluate the switch pattern of patients’ medical visits from general hospitals to CHCs after the policy reforms. A medical visit was counted if it was T2DM-related (with a T2DM diagnosis or prescription of diabetic medication) and occurred on any day following the policy’s release.

Two levels were used in the analysis: patient as a unit and visit as a unit. The total number of medical visits at each facility type in each policy period was used to evaluate the volume of the switch pattern. The data for a subset of patients who had at least one T2DM-related medical visit during each policy cycle were extracted for the patient level analysis. For the subgroup analysis, the primary time-to-event outcome for this investigation was the length (measured by days) to the first switch from a general hospital to a CHC during each policy period.

The time to the first switch from a CHC to a general hospital is illustrated below:

To evaluate policy period k, where k = 1, …, K, a *patient was counted as switching from a general hospital to a CHC if he/she made at least one visit to a CHC during P*_*k*_ *and the last visit in P*_*k-1*_ *was at a general hospital. The number of days from the start of P*_*k*_ *to the first CHC visit was counted as the time to the first switch.*

This switch pattern was used to evaluate the impact of the policies, including how many patients switched to CHCs and how quickly switching occurred following each policy change in this specific sub-population.

### Baseline covariates

The baseline covariates during the 360-day pre-index period in the analysis included age, gender, employment status, comorbidities, and insurance type. Patients with at least one comorbidity theoretically should have needed a greater care and fewer medical visits to CHCs. The comorbidities in the analysis included the following: hypertension; dyslipidemia; stroke; myocardial infarction (MI); acute complications of T2DM including diabetic ketoacidosis (DKA), hyperglycemia and osmotic pressure syndrome and diabetic lactic acidosis (DLA); chronic complications of diabetes mellitus including diabetic nephropathy, diabetic retinopathy and blindness, diabetic neuropathy, lower extremity arterial disease of diabetes patients (LEADDP), and diabetic foot; tumor or cancer; and special cases of diabetes mellitus including hypoglycemia. History of hospitalization, medication usage related to diabetes, hypertension, and dyslipidemia, as well as anti-platelet medication usage, were also included as covariates.

### Statistical analysis

Descriptive statistics for categorical variables included count and percentage, and for continuous variables included mean, standard deviation, and median. In bivariate analysis, the correlation between each policy variable and patient behavior change was calculated. Multivariate logistic regression was used to calculate adjusted odds ratio (OR) and 95% confidence intervals (CI) of a visit taking place in a CHC with adjustment for visit-specific covariates: age interval, sex, and pre-visit conditions during the 360-day pre-index period.

An initial k-means cluster analysis was performed to stratify patients by medical care-seeking behavior. Six features were used to build the distance: number of outpatient visits in non-general hospitals, number of outpatient visits in general hospitals, number of referrals up from CHCs to general hospitals, number of referrals down from general hospitals to CHCs, number of consecutive outpatient visits to a CHC, and number of consecutive outpatient visits to a general hospital. Characteristics of each cluster were evaluated (Table 4).

The KM curves were generated using days from each policy change until patients’ first switch from a general hospital to a CHC. Estimates were stratified by policy period. Patients were censored at the end of each policy period. All *p*-values were two-sided and *p*-value <0.05 was considered statistically significant. All statistical analyses were performed using SPSS version 23 and R 3.3.1.

## Results

**Fig. 2:**
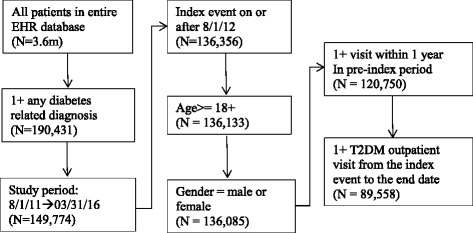
Patient flow diagram

Medical records from 503 CHCs and 63 general hospitals in Xiamen were included in the EHR database during the study period. The database included 190,431 individuals who had at least two T2DM-related visits during the study period. After applying the inclusion and exclusion criteria, we established an open cohort with 89,558 T2DM patients (Fig. 2). Of the included patients, 49,965 (55.8%) were newly-reported cases during the study period.

Baseline demographic characteristics were similar across the policy periods (Table 2). About 90% of the patients were older than 40 years. The share of patients enrolled in the New Rural Cooperative insurance plan significantly increased from 7.6% in 2012 to 13.3% in 2016. The comorbidity of hypertension and dyslipidemia were high, affecting 73.7% and 56.1% of cohort members respectively. Among cohort members, 27.0% had been diagnosed with stroke and 49.0% had been diagnosed with a myocardial infarction.

**Table 2:** Demographic characteristics of T2DM patients by policy period (%)

	Overall	P_1_	P_2_	P3	P_4_	P_5_	P_6_
	(*n* = 89,558)	(*n* = 59,274)	(*n* = 52,041)	(*n* = 46,861)	(*n* = 38,352)	(*n* = 50,888)	(*n* = 38,810)
Gender							
Male	46·0%	45·6%	45·6%	46·6%	46·6%	46·4%	47·1%
Female	54·0%	54·4%	54·4%	53·4%	53·4%	53·6%	52·9%
Age (years)							
18–39	10·1%	8·9%	7·1%	6.0%	5·5%	5·5%	4·8%
40–64	59·4%	59·3%	57·7%	56·5%	56·1%	56·2%	56·1%
65+	30·5%	31·8%	35·2%	37·5%	38·4%	38·3%	39·1%
Employee status							
Yes	89·6%	90·3%	91%	91·4%	91·7%	91·7%	91·8%
No	10·4%	9·7%	9.0%	8·6%	8·3%	8·3%	8·2%
Insurance plan type							
Urban employee	27·3%	28·5%	29·2%	29.0%	29·4%	28·1%	28·5%
Urban residence	48·7%	51.0%	50·2%	49·4%	48·2%	48·6%	48·3%
New rural cooperative	12·4%	9·8%	10·4%	11·7%	12·5%	13·2%	13·3%
Others	11.6%	10.7%	10.2%	9.9%	9.9%	10.1%	9.9%
Complications of interest							
Hypertension	73·7%	78·1%	77·0%	75·8%	75·1%	74·8%	73·6%
Dyslipidemia	56·1%	61·6%	60.0%	58·2%	57·9%	56·5%	56·7%
Stroke	27·0%	31·6%	31·7%	30·2%	30·5%	28·2%	29·3%
Myocardial infarction	49·0%	55·4%	54.0%	51·7%	51·1%	49·3%	49·8%
Chronic complications	16·2%	21·5%	21·5%	21·3%	22·2%	19·3%	21·2%
Acute complications	0·48%	0·20%	0·18%	0·17%	0·07%	0·13%	0·09%
Tumor or cancer	17·6%	19·4%	19.0%	18·1%	17·7%	16·7%	17.0%
Hypoglycemia	0·36%	0·18%	0·13%	0·08%	0·11%	0·09%	0·05%

**Fig. 3:**
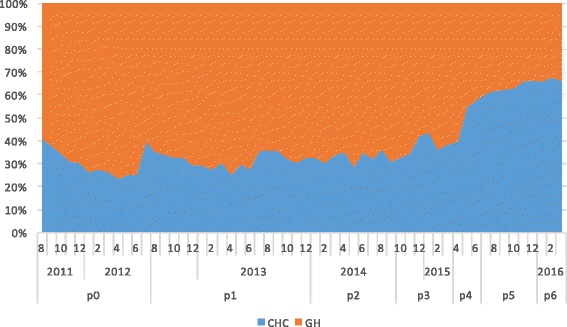
Volume changes of medical visits to general hospitals and CHCs between policies

In total, 2,373,524 T2DM-related medical visits were made during the study period among which 60.7% were to general hospitals. As shown in Fig. 3, the proportion of visits made to CHCs increased from 29.7% in 2012 to 66.5% by the end of evaluation period in 2016. The odds of a medical visit to a CHC significantly increased during the study period, especially for policy periods P_4−_ P_6_, when the comprehensive “three-in-one” team-based care model began to be fully implemented in Xiamen (Table 3). The odds of a visit to a CHC decreased significantly among patients with complications, suggesting that the patients most affected by the policy changes were those with stable T2DM.

**Table 3:** Multivariate logistic regression of the medical visits to CHCs

	Two-sided *p*-value	OR	95% CI	
			Lower	Upper
Gender				
Female (ref.)		1·0		
Male	<0.001	0·841	0·836	0·845
Age group, years				
18–39 (ref.)		1·0		
40–64	<0.001	2·798	2·755	2·843
65+	<0.001	3·330	3·276	3·384
Policy period				
P_0_ (ref.)		1·0		
P_1_	<0.001	1·111	1·102	1·121
P_2_	<0.001	1·247	1·236	1·259
P_3_	<0.001	1·580	1·564	1·597
P_4_	<0.001	2·809	2·773	2·845
P_5_	<0.001	4·745	4·696	4·794
P_6_	<0.001	5·675	5·599	5·752
If newly reported cases				
Yes v.s. No.	<0.001	1·623	1·600	1·647
Employee status				
Yes v.s. No.	<0.001	1·176	1·161	1·191
Insurance plan type				
New rural cooperative	<0.001	1·186	1·169	1·204
Urban residence	<0.001	1·280	1·265	1·295
Urban employee	<0.001	1·282	1·266	1·297
Others (ref.)		1·0		
Complications of interest				
Hypertension	<0.001	1·109	1·100	1·117
Dyslipidemia	<0.001	0·826	0·820	0·831
Stroke	<0.001	0·701	0·696	0·706
Myocardial infarction	<0.001	0·838	0·832	0·844
Chronic complications	<0.001	0·431	0·428	0·434
Acute complications	<0.001	0·283	0·263	0·304
Tumor or cancer	<0.001	0·828	0·822	0·835
Hypoglycemia	<0.001	0·607	0·569	0·647

**Fig. 4:**
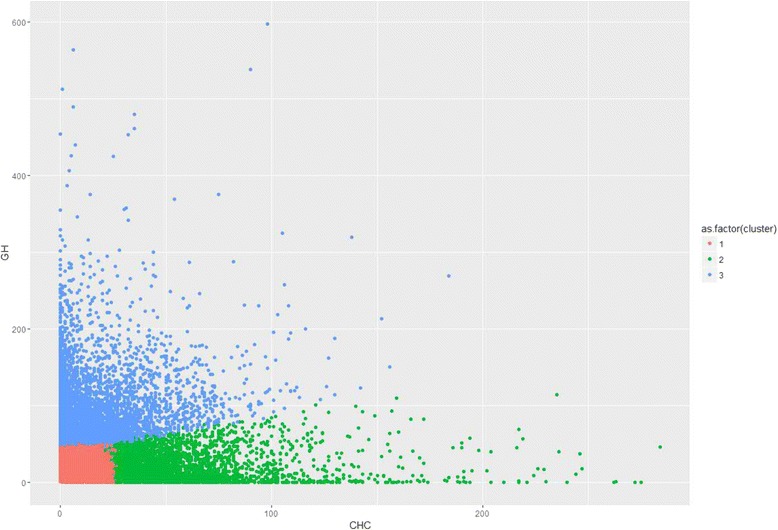
Three clusters identified by the K-means cluster analysis

**Fig. 5:**
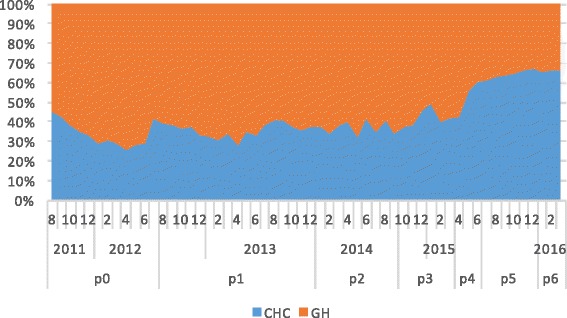
Switch patterns per policy for inactive patients (cluster 1)

**Fig. 6:**
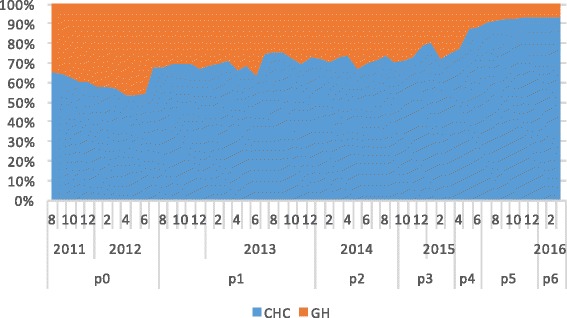
Switch patterns per policy for patients primarily visiting CHCs (cluster 2)

**Fig. 7:**
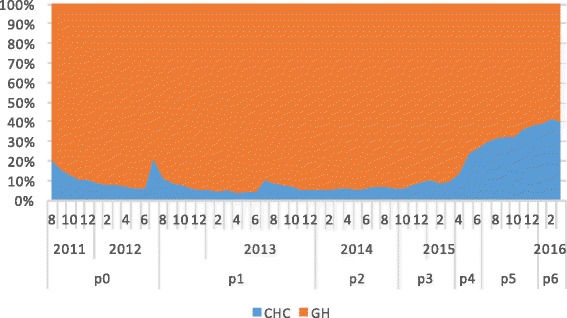
Switch patterns per policy for patients primarily visiting general hospitals (cluster 3)

Three patient groups were identified by the k-means cluster analysis. From the feature distribution of each cluster, we observed that Cluster 1 represented inactive patients who made very few T2DM-related outpatient visits to either general hospitals or CHCs, Cluster 2 represented patients who made frequent outpatient visits to general hospitals, and Cluster 3 represented patients who made frequent outpatient visits to CHCs (Fig. 4). The major difference among the three clusters was the prevalence of complications (Table 4). However, the three clusters had the same patterns of response to the policy changes (Figs. 5, 6 and 7). Further analysis is needed to understand the additional factors associated with the different medical care-seeking patterns of the different groups.

**Table 4:** Demographic characteristics of T2DM patients by clusters

	Overall	Cluster 1	Cluster 2	Cluster 3
	(n = 89,558)	(*n* = 75,516)	(*n* = 7558)	(*n* = 6484)
Gender				
Male	46·0%	46·1%	45·6%	44·9%
Female	54·0%	53·9%	54·4%	55·1%
Age (years)				
18–39	10·1%	11·4%	2·1%	4·1%
40–64	59·4%	59·7%	59·1%	56·2%
65+	30·5%	28·9%	38·8%	39·7%
Employee status				
Yes	89·6%	88·8%	95·2%	92·3%
No	10·4%	11·2%	4·8%	7·7%
Insurance plan type				
Urban employee	27·3%	26·3%	32·8%	31·4%
Urban residence	48·7%	48·1%	51·2%	52·2%
New rural cooperative	12·4%	13·2%	9·8%	6·9%
Others	11.6%	12.4%	6.2%	9.5%
Complications of interest				
Hypertension	73·7%	71·5%	83·1%	88·5%
Dyslipidemia	56·1%	52·9%	67·4%	80·3%
Stroke	27·0%	23·6%	36·5%	55·1%
Myocardial infarction	49·0%	45·4%	62·8%	75·0%
Chronic complications	16·2%	11·9%	25·6%	54·7%
Acute complications	0·48%	0·5%	0·8%	1·5%
Tumor or cancer	17·6%	16·9%	17·9%	25·9%
Hypoglycemia	0·36%	0·4%	0·5%	1·0%

**Fig. 8:**
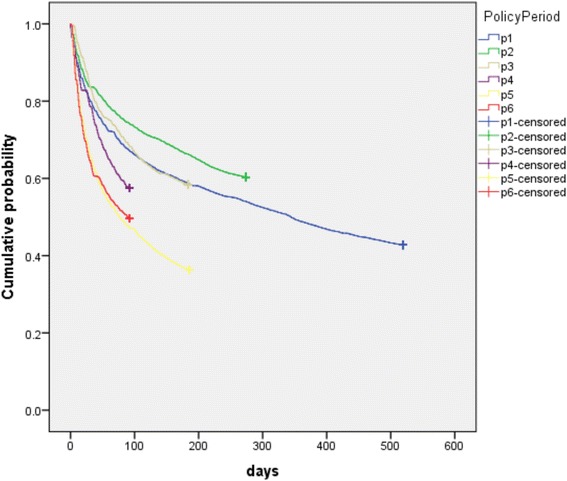
Kaplan-Meier (KM) curves of days from policy change until patients’ first switch from general hospital to CHC

In order to investigate the speed of behavior change following each new policy, we also conducted a patient-level analysis of the days from the start of each policy period until the first switch from the general hospital to CHC. Similar to the results were found in medical visit volume, patient switching accelerated most during policy periods P_4−_ P_6_ (Fig. 8).

## Discussion

### Summary of main findings

As a pilot city for health policy reform, Xiamen policy-makers redesigned the health care system for chronic disease management centered on a “three-in-one” team-based care model. In addition, they also implemented several other policy changes which included: increasing medication availability in CHCs (expanding the EDL to CHCs and extending duration per prescription) and providing financial incentives for providers (establishing and increased fees for chronic disease case management and enhanced reimbursement payment mechanism). Our study showed that the “three-in-one” team-based care model along with those policy reform measures had produced the desired effect in channeling more patient visits from GHs to CHCs, the proportion of visits to CHCs increased from 29.7% in 2012 to 66.5% in March 2016. This was a remarkable achievement in the backdrop of the national and provincial trends: the proportion of outpatient visits to CHCs decreased from 26.4% in 2013 to 24.8% in 2015 for the country, and from 24.3% in 2013 to 22.7% in 2015 for Fujian Province, based on the available data from the NHFPC Statistics and Information Center [13, 14].

Furthermore, the study also showed the speed of the shift in the proportion of visits from GHs to CHC had accelerated in the later part of the study period, particularly during Periods P_4_ to P_6_ in 2015–2016, as shown by the odds rations in Table 4 for P_4_ to P_6_ (2.8, 4.8 and 5.7, respectively), the areas under the curves for CHCs in Figs 3, 5,6 and 7 for P_4_ to P_6_, and the slopes of the KM curves in Fig. 8 for P_4_ to P_6_. Considering there were multiple policy measures and each was adjusted and improved over time based on the feedback and observation of the outcomes during the implementation, the study findings suggested that (1) it takes time for policy measures to take effect, and (2) policy makers need to be sensitive to feedback and flexible to adjust and modify the policy measures in order to achieve their desired effects.

Another important finding was that older patients and those with more difficult-to-treat complications made more visits to GHs than those younger patients or those without the complications, as shown by the odds ratios in Table 4. Because visits to GHs should be based on referrals from the “three-in-one” teams, this finding suggested the “three-in-one” teams-based care model started to function as designed.

### Learnings

This study may help healthcare policy makers in other cities and regions see the value of the “three-in-one” team-based care model for NCD management. First, this model may improve the efficiency of healthcare system by mobilizing resources in CHCs to handle more routine outpatient visits and case managements, to free up resources in hospitals to focus on more critical care for more difficult-to-treat complications, and to reduce the overall costs for NCD management. Second, the “three-in-one” team structure allows the three types of health care workers in the teams to maximize their time and clinical expertise in efficiently managing NCD patients: the specialists from hospitals may cover more patients with the support of CHC-based GPs, who in turn may improve their expertise with the assistance of specialists from tertiary hospitals. As is the case in many developed countries, health managers maintain the continuity and improve the effectiveness of patient care via frequent contacts with patients and providing additional services like health education, lifestyle interventions and follow-ups, and increasing the efficiency of case management by reducing the workload from the specialists and GPs. To sustain and improve the effect of the “three-in-one” team-based care model for chronic disease treatment and management, the roles of GPs and health managers should be further increased through standardized professional training and performance assessment. The “three-in-one” team-based care model’s effectiveness can be further improved by the use of advanced information technology for patient management, such as the guideline-based Clinical Decision Support System (CDSS) being piloted in Xiamen.

In addition to the creation of “three-in-one” teams, this study also demonstrated that successful shifting of patient care from GHs to CHCs also depends on other policy changes including an appropriate level of CHC staffing by GPs and health managers trained in NCD management, financial incentives for health care personnel, and adequate supplies of medications in CHCs from an expanded essential drug list.

Finally, the “three-in-one” model may be effective not only for the treatment of diabetes and hypertension, but for the related NCDs, many of which have the same risk factors and similar methods of control and prevention. Therefore, the application of diabetes management strategies to other NCDs may be cost-effective for the healthcare system.

### Strengths and limitations of the study

The major strength of this study was the use of real-world evidence based on EHRs to evaluate the effect of health care system reform in a large Chinese city. The effect of health reforms in China has been much debated. However, the overall effect of a major health system reform was often unmeasured, restricting opportunities for learning about what works, what does not, and what factors affect success [15].

Evaluation of large-scale reform to improve public health has always been a challenge for policy makers, especially how to measure policy effectiveness. Traditional designs have been dominated by experimental approaches used in medical research, in which a specific intervention is evaluated by comparing individuals or clusters of people in areas with and without the intervention. Such methods often may not be feasible when the new policies are being scaled up gradually. Policy implementation is also affected by many other factors that are often difficult to control in the experiments [16].

In this study, we used the regional EHR database in Xiamen, which provided complete information about the medical visits of more than 90% of Xiamen residents during the study period. This made it possible to perform a comprehensive evaluation of the effectiveness of health policy changes at the population level. In addition, the EHR platform itself formed a key component of the “three-in-one” team-based care model by allowing GHs and CHCs to share patient information and coordinate care.

Several limitations of the study should be recognized. Firstly, this study was limited by the lack of a top-level evaluation designed before policy implementation. This necessitated the retrospective collection of information about policy changes. Secondly, clinical data quality and completeness were also challenging. There was limited information about medical outcomes and lab test results, making it difficult to evaluate the quality of health care directly. Moreover, a comparison between patients in Xiamen and in other cities where the policy was not being implemented was not done side-by-side. Such data are expected to be considered in future studies.

## Conclusion

Health care reform in Xiamen, including the implementation of the “three-in-one” team-based care model, significantly increased the proportion of outpatient visits to CHCs by patients with T2DM.The study also demonstrates the feasibility of using real-world health data for evidence-based policy evaluation in China.

## Abbreviations

CDSS: Clinical decision supporting system
CHC: Community health center
CHCs: Community health centers
DKA: Diabetic ketoacidosis
DLA: Diabetes lactic acidosis
EDL: Essential drug list
EHR: Electronic health record
FPG: Fasting plasma glucose
GHs: General hospitals
GP: General practitioner
HbA1c: Hemoglobin A1c
KM: Kaplan-Meier
LEADDP: Lower extremity arterial disease of diabetes patients
MI: Myocardial infarction
NCD: Non-communicable diseaseNHFPC: National Health and Family Planning Commission
OR: Odds ratio
T2DM: Type 2 diabetes mellitus
